# Splicing Factor Transformer-2β (Tra2β) Regulates the Expression of Regulator of G Protein Signaling 4 (RGS4) Gene and Is Induced by Morphine

**DOI:** 10.1371/journal.pone.0072220

**Published:** 2013-08-19

**Authors:** Shu-Jing Li, Ya Li, Shi-chao Cui, Yao Qi, Jing-Jing Zhao, Xiao-Yan Liu, Ping Xu, Xian-Hua Chen

**Affiliations:** 1 State Key Laboratory of Medical Neurobiology and Department of Neurobiology,School of Basic Medical Sciences, Shanghai Medical College of Fudan University, Shanghai, China; 2 Laboratory of Genomic Physiology and Institutes of Brain Science, Shanghai Medical College of Fudan University, Shanghai, China; Rutgers University, United States of America

## Abstract

Regulator of G protein signaling 4 (RGS4) is a critical modulator of G protein-coupled receptor (GPCR)-mediated signaling and plays important roles in many neural process and diseases. Particularly, drug-induced alteration in RGS4 protein levels is associated with acute and chronic effects of drugs of abuse. However, the precise mechanism underlying the regulation of RGS4 expression is largely unknown. Here, we demonstrated that the expression of RGS4 gene was subject to regulation by alternative splicing of the exon 6. Transformer-2β (Tra2β), an important splicing factor, bound to RGS4 mRNA and increased the relative level of RGS4-1 mRNA isoform by enhancing the inclusion of exon 6. Meanwhile, Tra2β increased the expression of full-length RGS4 protein. In rat brain, Tra2β was co-localized with RGS4 in multiple opioid action-related brain regions. In addition, the acute and chronic morphine treatment induced alteration in the expression level of Tra2β in rat locus coerulus (LC) in parallel to that of RGS4 proteins. It suggests that induction of this splicing factor may contribute to the change of RGS4 level elicited by morphine. Taken together, the results provide the evidence demonstrating the function of Tra2β as a new mediator in opioid-induced signaling pathway via regulating RGS4 expression.

## Introduction

Regulators of G protein signaling (RGS) proteins are critical modulators of G protein-coupled receptor (GPCR)-mediated signal transduction [1]. These proteins act as GTPase-activating proteins (GAPs) for heterotrimeric Gα subunits, accelerating the shut-off mechanism for G protein signaling [2]. As one of the most extensively studied RGS proteins, RGS4 attenuates the intensity and duration of Gαi/o and Gαq/11 subunits-coupled receptor signaling [3], [4] and is involved in many clinical diseases. Microarray and genomic analyses showed decreased levels of RGS4 in the prefrontal cortex in patients with schizophrenia [5]. Meanwhile, polymorphisms of the RGS4 gene have been identified in schizophrenia patients [6]. Genetic studies indicate RGS4 as a vulnerability factor for schizophrenia [5], [7]. In addition, RGS4 plays important roles for dopaminergic control of striatal long-term depression and susceptibility to Parkinson’s disease [8] as well as in neural plasticity [9].

RGS4 is highly expressed in the brain and robustly distributed in regions that are involved in drugs of abuse-induced response and cognition processes. These regions include the prefrontal cortex, striatum, hippocampus and locus coeruleus [10]–[12]. The expression pattern of RGS4 is well consistent with recent studies showing the importance of RGS4 for psychostimulant and opiate drug-induced actions in the brain. Several *in vitro* systems have linked RGS4 to the regulation of μ-opioid receptor signaling [13]–[15]. Studies of RGS4 knockout mice also support a role of RGS4 in morphine reward and physical dependence [16].

In view of the role of RGS4 in regulation of μ-opioid receptor signaling, we could reasonably expect that a dynamically tight control of RGS4 protein levels would be critical for RGS4 protein to regulate the duration and/or intensity of GPCR signaling in response to opioid administration. Thus, drug-induced changes in RGS4 protein levels could contribute to the acute responses and chronic effects of tolerance, dependence and sensitization. Indeed, there is evidence that RGS4 is highly regulated not only by various physiological stimuli such as nerve growth factor [17], stress and corticosteroids [18], but also is selectively induced by drugs of abuse in different brain regions [19]–[22]. Those drug-induced changes in RGS4 levels have been linked to its roles in acute dopaminergic responses as well as chronic drug-induced behaviors [16], [19]–[21].

In contrast to the physiological importance of tightly controlled RGS4 expression, the regulatory mechanisms of RGS4 expression under opioid treatment remain unknown. Four splice variants of RGS4 gene have been described in human brain, among which RGS4-1 (NM_005613.3, variant 1 & 2 in Ding et al., 2007) and RGS4-4 (AK093959, variant 4 in Ding et al., 2007) are the most abundant splice variants in human brain [23]. Variants RGS4-1 and RGS4-4 differ by alternative usage of the exon 6. RGS4-1 variant includes the exon 6 and is highly conserved between human and rodents. It encodes the 205-amino acid RGS4 protein which contains the entire RGS domain. The RGS domain is responsible for the GTPase-activating protein (GAP) activity that negatively regulates G protein function [2]. RGS4-4 isoform excludes the exon 6, encoding a 93-amino acid protein with a truncated RGS domain. This truncated form is nonfunctional [23]. Thus, the alternative splicing of the exon 6 is critical for regulation of RGS4 function. The expression of RGS4 isoforms depends on tissue-type, development stage and pathological condition [23]–[26]. It is likely that alternative splicing may play an important role in regulation of RGS4 expression. However, the experimental evidence for this likelihood remains to be demonstrated.

Pre-mRNA alternative splicing enables the generation of proteins with different functions and structures through variations in the splicing patterns of pre-mRNA from one gene. The pre-mRNA splicing takes place in the spliceosome, a large RNA-protein complex [27]. The assembly of the spliceosome involves a series of RNA-RNA, RNA-protein and protein-protein interactions [28], [29]. Serine/arginine-rich (SR) proteins are important components of the spliceosome. They modulate splice site selection and splicing efficiency through binding to specific RNA sequences and assembling the spliceosome at weak splice sites [30]. Here, we reported the role of Transformer-2β (Tra2β), a member of SR proteins, in regulation of RGS4 expression in the brain. In addition, we also characterized the function of Tra2β in the regulation of RGS4 expression *in vivo*, by determining the Tra2β expression in rat brain under opioid treatment. We found that the changes of Tra2β in rat LC were accompanied by the changes of RGS4 in acute and chronic morphine administration. The results suggest the role of Tra2β as a candidate regulator of RGS4 expression in opioid-induced signaling.

## Results

### Tra2β Regulates the Expression of RGS4 in Cultured Cells and in Rat LC

**Figure 1:**
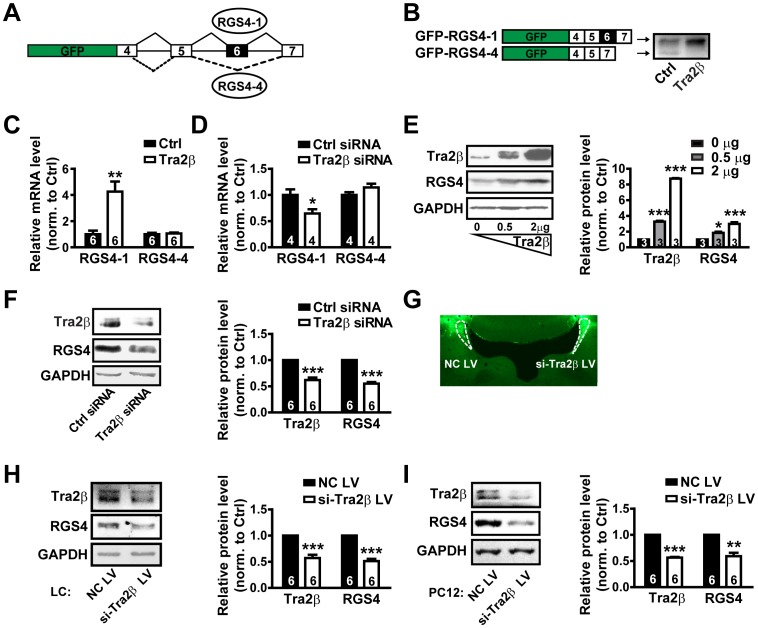
Effects of Tra2β over-expression and silence on RGS4 expression in SH-SY5Y cultured cells and rat LC. (A) An illustration of GFP-RGS4 minigene construction. The GFP reporter gene was fused in-frame with RGS4 genomic DNA sequences consisting of RGS4 exons 4, 5, 6 and 7. Exons are shown as boxes, introns as lines. (B) Tra2β over-expression increased the ratio of RGS4-1 isoform to RGS4-4. SH-SY5Y cells were co-transfected with the GFP-RGS4 minigene plus Tra2β over-expression plasmids (Tra2β) or empty vectors (Ctrl). The GFP-RGS4-1 (43 kDa) and GFP-RGS4-4 (38 kDa) proteins were detected by GFP antibody. The structures of the GFP-RGS4-1 and GFP-RGS4-4 variants were illustrated on the left. (C) Tra2β over-expression increased relative mRNA level (%GAPDH) of RGS4-1 isoform (4.26±0.77), but not RGS4-4 isoform (1.06±0.07, *p* = 0.67) in SH-SY5Y cells. (D) Tra2β RNAi reduced relative mRNA level (%GAPDH) of RGS4-1 isoforms (0.65±0.08), but not RGS4-4 isoforms (1.14±0.07, *p* = 0.15) in SH-SY5Y cells. (E) Relative protein level (%GAPDH) of RGS4 in SH-SY5Y cells transfected with increasing amounts of Tra2β over-expression plasmids. Numbers indicate the microgram of Tra2β over-expression plasmids used for transfection. *p<0.05, **p<0.01, ***p<0.001 compared to 0 µg group, using one-way ANOVA. (F) Tra2β RNAi decreased relative protein level (%GAPDH) of RGS4 (0.55±0.03) in SH-SY5Y cells. (G) Illustration of rat right LC infected with lentivirus expressing siRNAs against Tra2β (si-Tra2β LV), and left LC infected with negative control lentivirus (NC LV) under fluorescent microscope. (H) Infection of si-Tra2β LV reduced Tra2β (0.57±0.06) and RGS4 (0.51±0.04) protein levels (%GAPDH) in rat LC. (I) Infection of si-Tra2β LV reduced Tra2β (0.56±0.01) and RGS4 (0.59±0.07) protein levels (%GAPDH) in PC12 cells. The molecular weight of Tra2β, RGS4 and GAPDH are 40 kDa, 28 kDa and 38 kDa, respectively. Data are expressed as mean ± s.e.m. from at least three independent experiments; “*n*” as denoted inside bar graphs, represents the number of cultures, except for (H) where they represent the number of rats. *p<0.05, **p<0.01, ***p<0.001, using unpaired two-tailed Student’s *t*-tests for real-time qPCR experiments and paired *t*-tests for immunoblots.

To examine the effect of Tra2β on the exon 6 splicing, we used a GFP-fused minigene construct in which the sequence encoding green fluorescent protein (GFP) is followed in frame with RGS4 minigene sequence. The RGS4 minigene consists of RGS4 exon 6 flanked by adjacent introns and constitutive exons, as illustrated in Fig. 1A. This minigene construct was used to transfect SH-SY5Y cells, a human derived neuroblastoma cell line, and generated two products. The major products were GFP-RGS4-1, whereas the minor were GFP-RGS4-4 (Fig. 1B, left). The splicing pattern of this minigene is similar to that of endogenous RGS4 gene *in vivo* (data not shown). Co-transfection of the minigene construct with the DNA construct expressing Tra2β protein altered the splicing pattern of the minigene. Tra2β overexpression significantly increased the level of GFP-RGS4-1 and reduced the level of GFP-RGS4-4. The results suggest that Tra2β promotes the exon 6 inclusive splicing (Fig. 1B).

To determine the effect of Tra2β on the expression of endogenous RGS4 gene, we directly examined the levels of RGS4 mRNAs and proteins in SH-SY5Y cells that endogenously express RGS4 gene. Real-time qPCR analysis showed that the relative level of RGS4-1 variant increased dramatically in the cells with Tra2β overexpression, while the relative level of RGS4-4 variant had no obvious changes (Fig. 1C). Western blot analysis also showed that the level of RGS4 protein (translation product of RGS4-1) was up-regulated by overexpression of Tra2β in a concentration-dependent manner (Fig. 1E). In addition, when the expression of Tra2β was knocked-down by Tra2β-specific siRNAs, both the level of RGS4-1 mRNA (Fig. 1D) and RGS4 protein (Fig. 1F) were significantly decreased. In comparison, the level of RGS4-4 variant had no significant changes (Fig. 1D). The level of RGS4-4 translation product was too low to be detected by Western blot (Fig. S1). The effect of Tra2β on expression level of RGS4 seems to be selective, as suggested by the lack of effect of Tra2β overexpression or silence on mRNA levels of other RGS proteins as RGS9-1 and RGS9-2, two splice variants of RGS9 gene with different distribution and functions in the nervous system [31] (see Fig. S2). These results indicate that Tra2β upregulates the level of variant RGS4-1 and more importantly, increases the corresponding functional RGS4 protein level in cultured neuronal cells.

To further examine whether Tra2β regulates RGS4 expression *in vivo*, we studied the effect of Tra2β on RGS4 expression in rat brain. In the experiments, the lentivirus encoding siRNAs against Tra2β (si-Tra2β LV) or negative control lentivirus encoding scrambled siRNAs (NC LV) were stereotactically delivered into the right or left locus coeruleus (LC) of rat brain, respectively (Fig. 1G). Western blot results showed that the right LC infected with si-Tra2β LV had much lower levels of Tra2β proteins (43% of decrease) and RGS4 proteins (49% of decrease) compared to the left LC (Fig. 1H). Studies in rat PC12 cells also confirmed that si-Tra2β LV-mediated Tra2β knockdown resulted in decreased expression level of RGS4 (Fig. 1I). These results indicate that RGS4 expression is regulated by Tra2β *in vivo*.

### Tra2β Protein Interacts with RGS4 mRNAs

**Figure 2:**
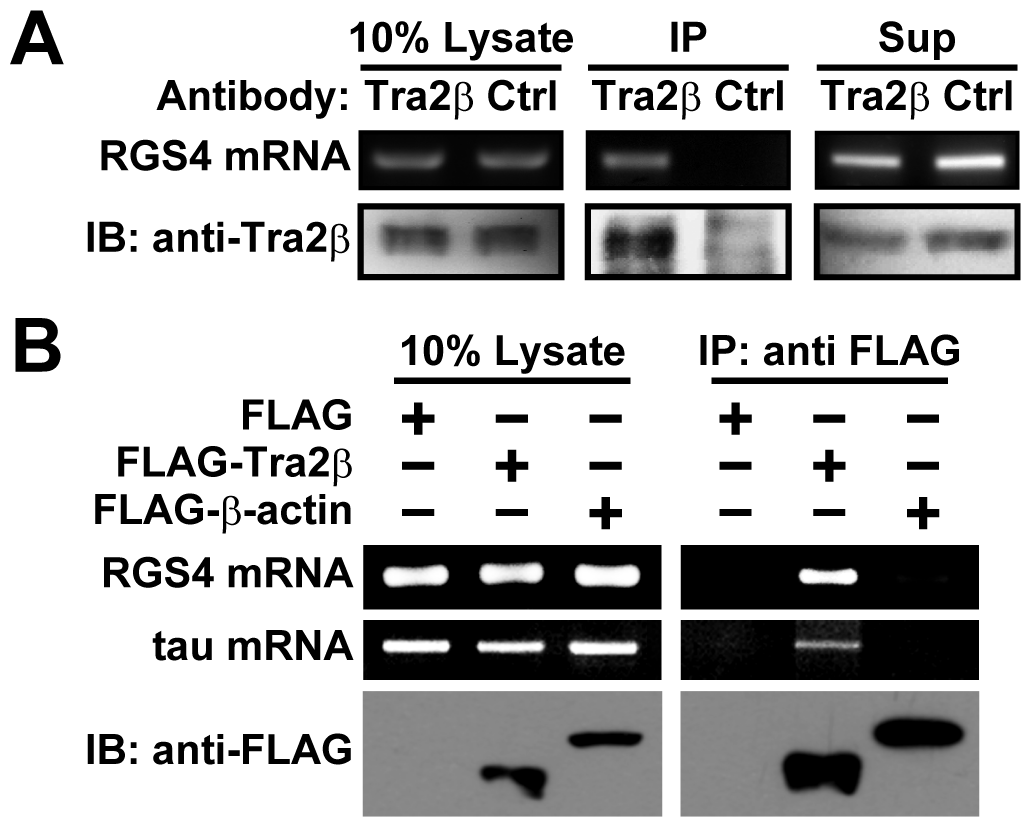
RGS4 mRNAs are co-immunoprecipitated with Tra2β protein. (A) Lysates extracted from whole rat brain were immunoprecipitated with anti-Tra2β antibody (Antibody: Tra2β) or control antibody (Antibody: Ctrl). The tissue lysate (10% Lysate), IP complex (IP) and 10% supernatant after IP (Sup) were analyzed for RGS4 mRNAs and Tra2β protein (40 kDa), respectively. IB: immunoblot. (B) Lysates extracted from SH-SY5Y cells transfected with plasmids expressing FLAG-Tra2β (41 kDa) or FLAG-β-actin (43 kDa, negative control) or FLAG alone (negative control) were immunoprecipitated with anti-FLAG monoclonal antibody-agarose conjugates. The cell lysate (10% Lysate) and IP complexes (IP: anti-FLAG) were analyzed for RGS4 mRNAs (tau mRNAs as a positive control) and FLAG-Tra2β (or FLAG-β-actin) proteins, respectively. All data are representative images from at least three independent experiments.

To investigate whether Tra2β regulates RGS4 expression via its binding to RGS4 mRNAs, we performed RNA-binding protein immunoprecipitation (RIP) assays to examine if RGS4 mRNAs are present in the immunoprecipitated complex of Tra2β. In the RIP assay, rabbit anti-Tra2β antibody and rabbit anti-GAP43 (negative control) were incubated with equal amount of rat brain lysates, then pulled down by Protein A sepharose beads, and the antibody-precipitated mRNAs and proteins were analyzed by RT-PCR and Western blot analyses, respectively. As shown in Fig. 2A, RGS4 mRNAs were precipitated by anti-Tra2β antibody, but not by the control antibody, suggesting an interaction between RGS4 mRNAs and Tra2β protein. In addition, we also examined the binding of recombinant Tra2β protein to RGS4 mRNAs in human SH-SY5Y cells. Anti-FLAG monoclonal antibody-agarose conjugates were incubated with the whole cell lysates from the cells transfected with plasmids expressing FLAG-Tra2β, FLAG-β-actin or FLAG plasmids. Results showed that RGS4 mRNAs were precipitated by FLAG-Tra2β, but not by FLAG-β-actin or FLAG alone (the controls). Tau mRNAs were detected as a positive control in this assay, because tau mRNAs have been reported to bind with Tra2β [32] (Fig. 2B).

### The SR Proteins ASF/SF2 and SRp30c Interact with Tra2β

**Figure 3:**
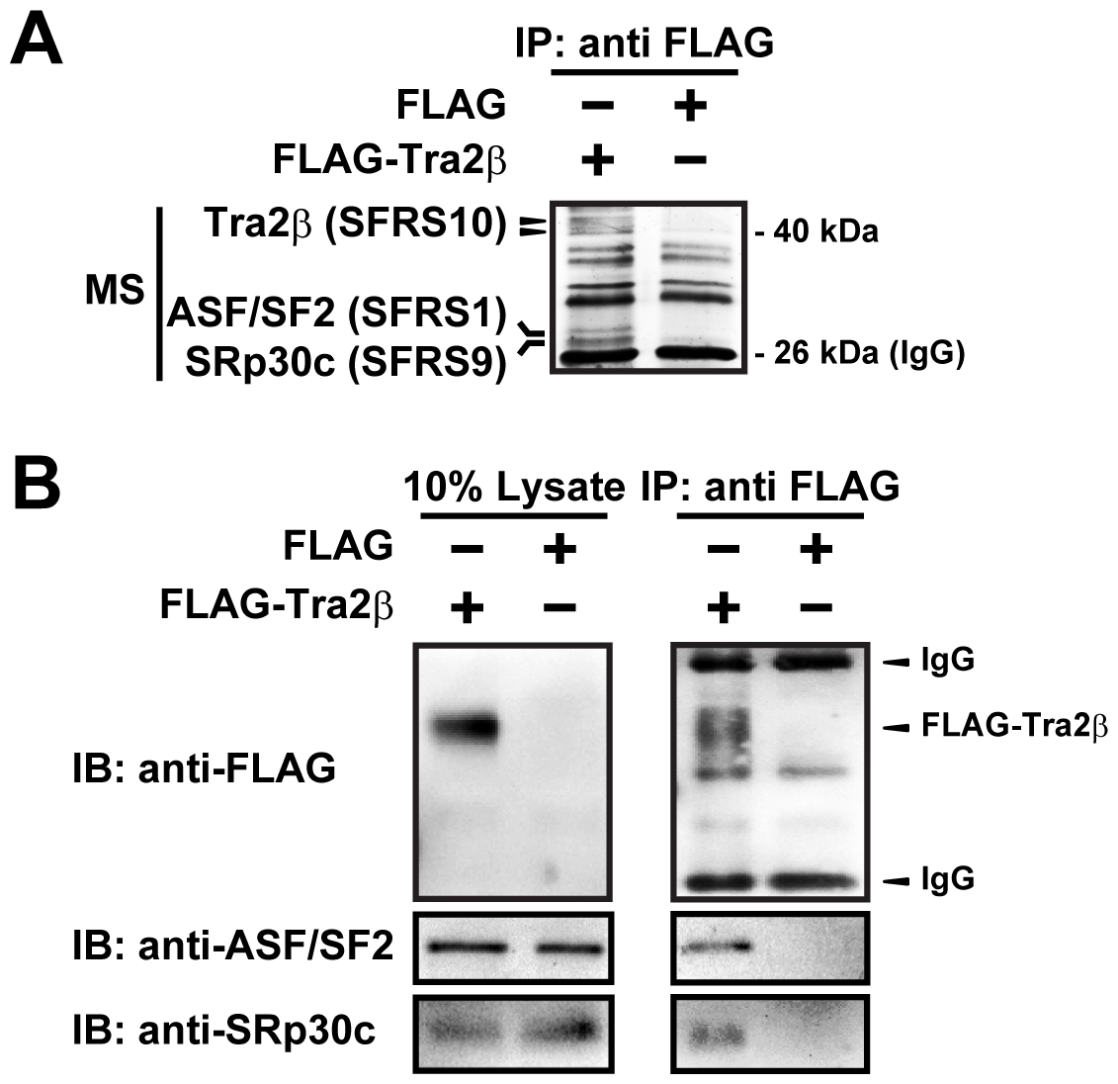
ASF/SF2 and SRp30c are co-immunoprecipitated with Tra2β protein. (A) IP complexes of FLAG-Tra2β (Tra2β) and FLAG (Ctrl) were analyzed by SDS-PAGE followed by silver staining. The additional bands (marked by the arrows and lines) in the IP complexes of FLAG-Tra2β compared with the control were cut from the gel and their identities were obtained by Mass Spectrometry analysis. (B) Detection of ASF/SF2 (32 kDa) and SRp30c (28 kDa) proteins in the IP complex of Tra2β using antibodies specific to ASF/SF2 and SRp30c. IB: immunoblot. All data are representative images from at least three independent experiments.

As an SR protein, Tra2β largely functions as a binding protein to its target mRNAs and also with other splicing factors, thereby contributing to spliceosome assembly and splicing site recognition [32], [33]. Here, we investigated the factors interacting with Tra2β protein by examining the precipitates of FLAG-Tra2β in whole cell lysates from SH-SY5Y cells transfected with plasmids expressing FLAG-Tra2β or FLAG plasmids. By silver staining of SDS-PAGE gel, two bands were found in FLAG-Tra2β pull-down products, but not in the control. Mass spectrometry (MS) analysis demonstrated that they were ASF/SF2 and SRp30c, both belonging to SR protein family (Fig. 3A). Western blot analysis with specific antibodies against ASF/SF2 and SRp30c further confirmed their presence in the immunoprecipitated complex of Tra2β (Fig. 3B). These results suggest that Tra2β can bind to RGS4 mRNA, and can interact with ASF/SF2 and SRp30c as well.

### Tra2β is Co-localized with RGS4 in Rat Brain

**Figure 4:**
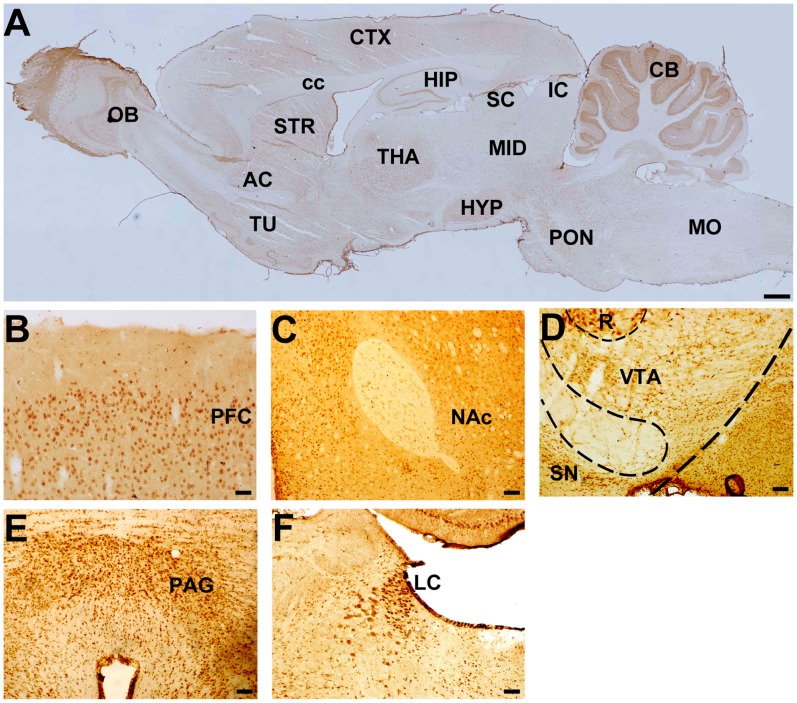
Distribution of Tra2β in adult rat brain. (A) Representative IHC images showing distribution of Tra2β proteins in the sagittal brain section. OB: olfactory bulb, TU: olfactory tubercle, CTX: cortex, HIP: hippocampus, STR: striatum, THA: thalamus, HYP: hypothalamus, SC: superior colliculus, IC: inferior colliculus, MID: midbrain, CB: cerebellum, PON: pons, MO: medulla oblongata, cc: corpus callosum. Scale bars: 600 µm. (B–F) Representative IHC images showing distribution of Tra2β proteins in coronal brain sections. PFC: prefrontal cortex, NAc: nucleus accumbens, VTA: ventral tegmental area, SN: substantia nigra, R: red nucleus, PAG: periaqueductal grey, LC: locus coeruleus. Scale bars: 80 µm.

RGS4 is widely expressed throughout the brain with the highest levels in the regions associated with the effects of drugs of abuse, such as the prefrontal cortex, striatum, hippocampus and locus coeruleus [10]–[12]. To explore the spatial correlation between Tra2β and RGS4 distribution *in vivo*, we performed immunohistochemical (IHC) staining and double immunofluorescence analysis with antibodies against Tra2β and RGS4. Tra2β immunoreactivity was found throughout the brain. However, the Tra2β immunosignal intensity is region-dependent. In the sagittal section of adult rat brain, Tra2β immunosignal intensity was strong in the olfactory bulb and cerebellum, moderate in the cerebral cortex, hippocampus, striatum and thalamus, and low in the superior colliculus, inferior colliculus and medulla oblongata (Fig. 4A). Interestingly, the detailed IHC analysis of coronal sections demonstrated that intense staining of Tra2β was observed in several brain regions implicated in the actions of drugs of abuse, such as the prefrontal cortex (PFC), nucleus accmbens (NAc), ventral tegmental area (VTA), periaqueductal grey (PAG) and locus coeruleus (LC), suggesting that Tra2β may play a role in opioid actions (Fig. 4B–F).

**Figure 5:**
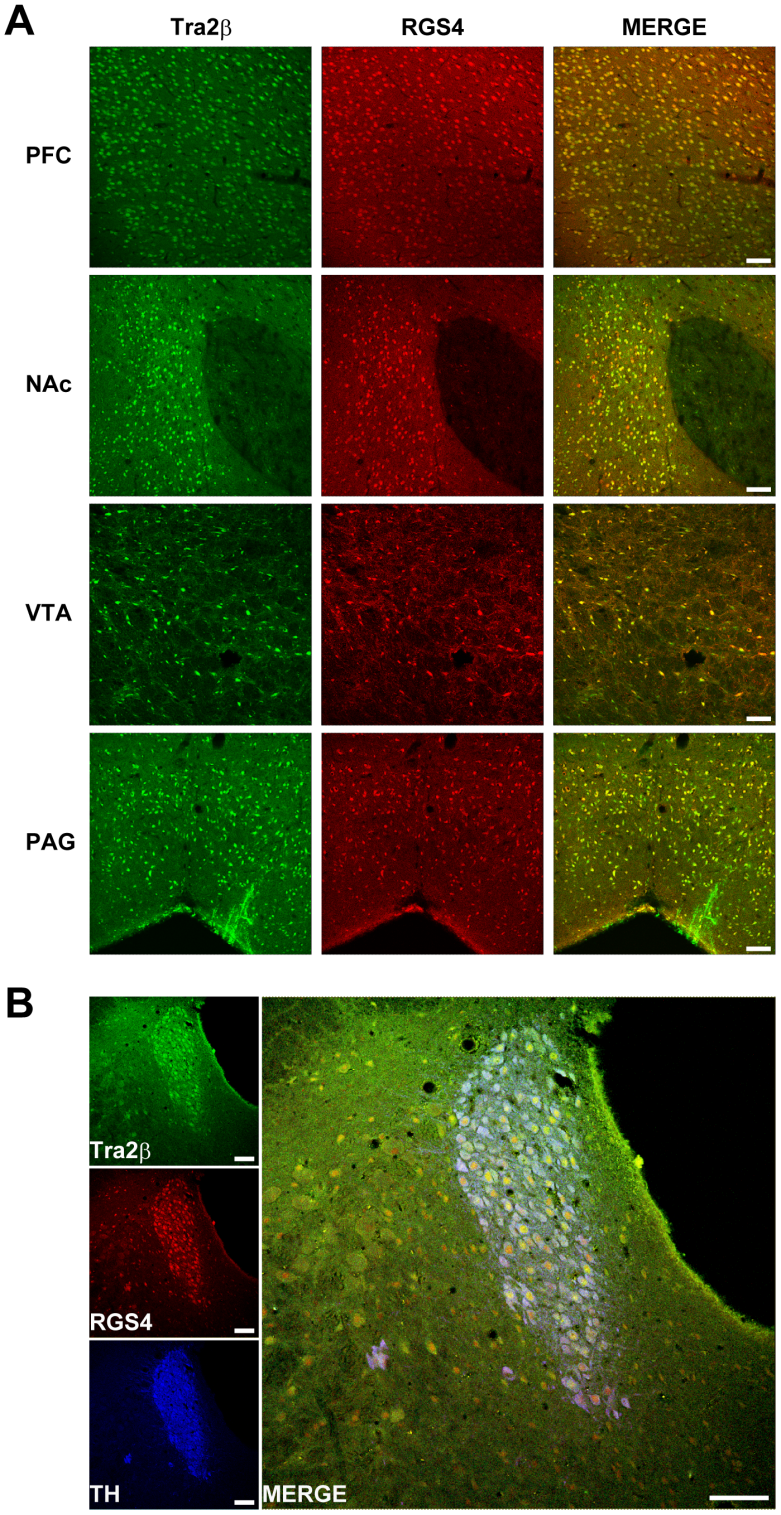
Co-localization of Tra2β and RGS4 in rat brain. (A) Representative immunofluorescence double-staining images of Tra2β (green), RGS4 (red) and overlay (MERGE). Scale bars: 80 µm. (B) Representative immunofluorescence triple-staining images of Tra2β (green), RGS4 (red), noradrenergic neuron marker TH (blue) and overlay (MERGE) in the LC. Scale bars: 80 µm.

By co-staining of the same brain sections with antibody against RGS4, we found that RGS4 immunoreativity was also strong in the regions with intense Tra2β immunosignals. When merged, images of the PFC, NAc, VTA, PAG (Fig. 5A) and LC (Fig. 5B) demonstrated a significant co-localization of Tra2β with RGS4. In addition, both Tra2β and RGS4 were expressed predominantly in neuron, but not in glia, as shown in Fig. S4. The distributional correlation in the brain further supports the potential functional relation between Tra2β and RGS4 *in vivo.*

### In Morphine-treated Rat LC, Both Tra2β and RGS4 Levels are Regulated in the Same Pattern

The findings described above indicate the possibility that Tra2β may be involved in alteration of RGS4 expression in vivo. Studies have shown that the expression of RGS4 is regulated by psychostimulants and opioids in the LC, a well-characterized brain region associated with opioid dependence and withdrawal. Acute morphine injection leads to a decrease in RGS4 protein level in the LC, whereas repeated morphine administration leads to an increase in RGS4 levels in the LC [19], [20]. To examine whether Tra2β contributes to the opioid-regulated RGS4 expression, we characterized the effect of morphine treatment on Tra2β and RGS4 levels in rat LC.

**Figure 6:**
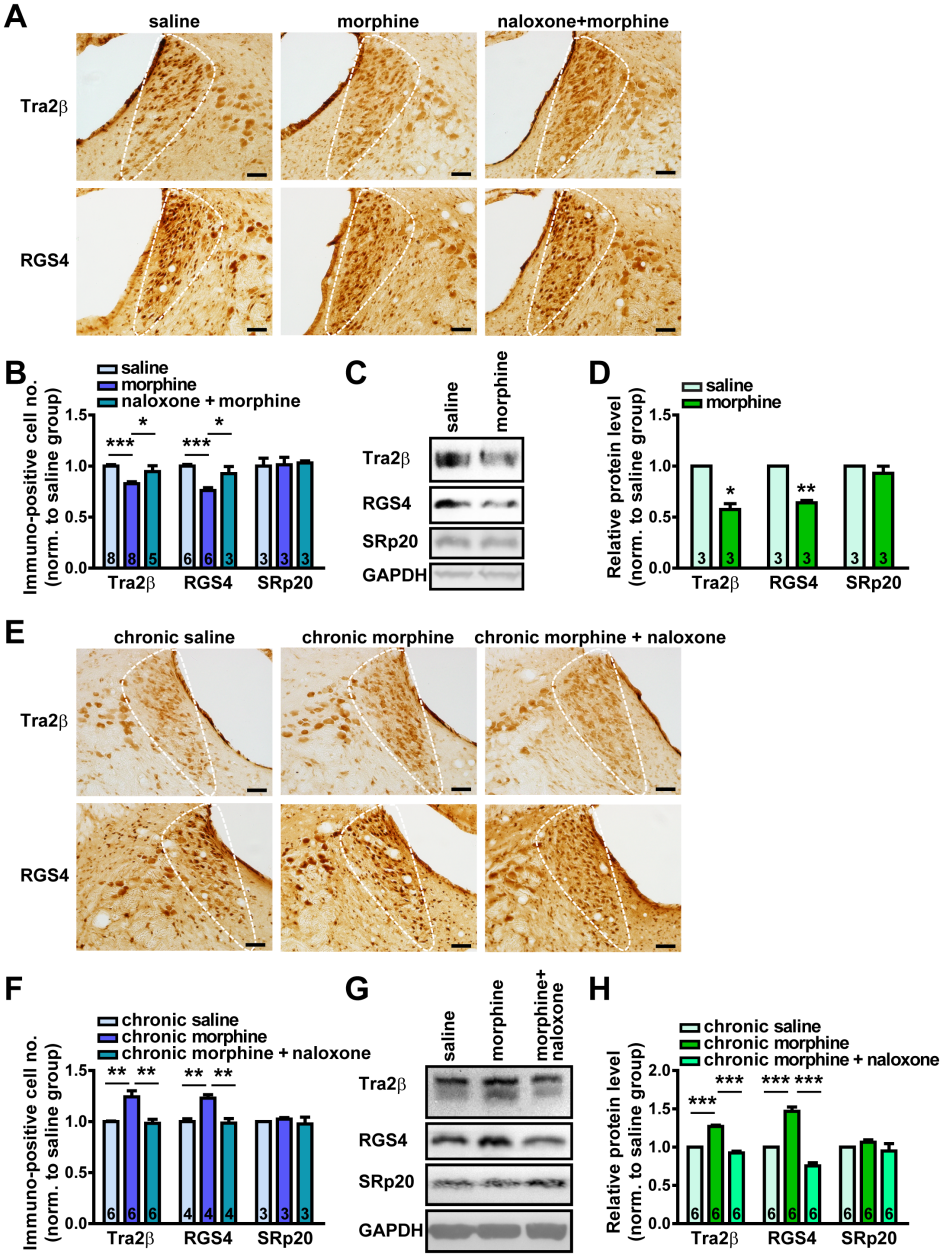
Regulation of RGS4, Tra2β and SRp20 levels in rat LC after acute and chronic morphine administration. (A) Representative IHC images of Tra2β and RGS4 in rat LC slices after acute saline, morphine and naloxone+morphine administration. Scale bars: 80 µm. (B) Quantitative analysis of Tra2β (acute saline, 1.00±0.01; acute morphine, 0.83±0.02; naloxone+morphine, 0.95±0.06), RGS4 (acute saline, 1.00±0.02; acute morphine, 0.76±0.03; naloxone+morphine, 0.93±0.07) and SRp20 (acute saline, 1.00±0.08; acute morphine, 1.01±0.07; naloxone+morphine, 1.03±0.02) positive cell number per cubic millimeter in rat LC slices. (C–D) Representative immunoblot images (C) and data statistics (D) showing the relative protein level (%GAPDH) of Tra2β, RGS4 and SRp20 in rat LC after acute morphine administration, normalized to that of acute saline group (Tra2β, 0.57±0.06; RGS4, 0.64±0.02; SRp20, 0.93±0.07; *p<0.05, **p<0.01, ***p<0.001, compared to saline group, using paired *t*-tests). (E) Representative IHC images of Tra2β and RGS4 in rat LC slices after chronic saline, chronic morphine and naloxone following chronic morphine administration. Scale bars: 80 µm. (F) Quantitative analysis of Tra2β (chronic saline, 1.00±0.01; chronic morphine, 1.24±0.06; chronic morphine+naloxone, 0.98±0.04), RGS4 (chronic saline, 1.00±0.03; chronic morphine, 1.23±0.03; chronic morphine+naloxone, 0.99±0.04) and SRp20 (chronic saline, 1.00±0.01; chronic morphine, 1.02±0.01; chronic morphine+naloxone, 0.98±0.07) positive cell number per cubic millimeter in rat LC slices. (G–H) Representative immunoblot images (G) and data statistics (H) showing the relative protein level (%GAPDH) of Tra2β, RGS4 and SRp20 in rat LC after chronic morphine and naloxone following chronic morphine administration, normalized to that of chronic saline group (Tra2β, chronic morphine, 1.27±0.02, chronic morphine+naloxone, 0.92±0.02; RGS4, chronic morphine, 1.45±0.06, chronic morphine+naloxone, 0.75±0.04; SRp20, chronic morphine, 1.06±0.03, chronic morphine+naloxone, 0.95±0.06; *p<0.05, **p<0.01, ***p<0.001, using paired *t*-tests). The molecular weight of Tra2β, RGS4, SRp20 and GAPDH are 40 kDa, 28 kDa, 20 kDa and 38 kDa, respectively. Data are expressed as mean ± s.e.m. from at least three independent experiments; “*n*” as denoted inside bar graphs, represents the number of rats. *p<0.05, **p<0.01, ***p<0.001, using one-way ANOVA followed by Tukey’s multiple-comparison test, unless otherwise specified.

By quantitative analysis of the number of Tra2β and RGS4 immuno-positive cells in the brain slices, we investigated whether acute morphine injection elicits alteration in Tra2β in rat LC. The results showed that the number of Tra2β positive cells was decreased significantly at 3 hours after a single morphine injection. The decrease was prevented by administration of opioid receptor antagonist naloxone before morphine injection (Fig. 6A–B). RGS4-positive cell number in LC was also reduced by acute morphine administration, which is consistent with previous findings [20]. To further confirm these findings, dissection of rat LC tissue and Western blot analysis of the total protein lysates were carried out. The results also revealed that the expression of Tra2β and RGS4 was down-regulated by acute morphine treatment (Fig. 6C–D).

We also observed the effects of chronic repeated morphine administration on the expression of Tra2β and RGS4. The results showed that the number of Tra2β positive cells and RGS4 positive cells in the LC were increased significantly after 14 days’ morphine administration when compared to the saline administration group. Besides, at day 14, naloxone-precipitated withdrawal resulted in marked decreases in both Tra2β and RGS4 protein levels 1 hour following injection of naloxone. These results indicated that the expression of Tra2β and RGS4 in the LC were regulated by chronic morphine treatment and naloxone withdrawal (Fig. 6E–F). Western blot analysis also confirmed that the expression of Tra2β and RGS4 was up-regulated by chronic morphine treatment and down-regulated by naloxone-precipitated withdrawal in rat LC (Fig. 6G–H). Meanwhile, neither acute nor chronic morphine administration had noticeable effect on the level of SRp20, another member of SR protein family, suggesting that the regulation of Tra2β by morphine is specific (Fig. 6). Therefore, no matter in acute or chronic administration, morphine elicits paralleled changes of Tra2β and RGS4 expression. Moreover, taking into account the findings in rat LC and cultured cells that Tra2β promotes the RGS4 expression, it is likely that Tra2β contributes to regulation of RGS4 expression in response to opioid treatment *in vivo*.

## Discussion

As important signal mediators in opioid action, RGS proteins play crucial roles not only in terminating acute opioid agonist action but also in opioid receptor desensitization, internalization, recycling, and degradation, thereby affecting opioid tolerance and dependence [1], [34]. Although RGS4 is an important component of opioid signaling, the mechanisms regulating expression of RGS4 gene have not been well elucidated. There are some reports about the transcription level and protein stability of RGS4, but little is known about the post-transcriptional regulation of RGS4 expression [26], [35]. The present study provides new evidence indicating that alternative splicing factor Tra2β-controlled changes in RGS4 mRNA might contribute to the drug-induced regulation of RGS4 levels.

Tra2β, a member of serine/arginine (SR)-rich protein family, is characterized by the RS domain rich in arginine and serine residues and it is heavily phosphorylated. Our previous work reported that the expression pattern of Tra2β was regulated in a tissue- and temporal-specific pattern in the developing human brain [36]. Several studies have demonstrated that proper concentration of Tra2β is important for normal cellular function [37]–[40]. In mammalian brain, a change of Tra2β concentration is concomitant with hypoxia [41], nerve injury [42] and Alzheimer’s disease [38]. Our findings demonstrate a new role of Tra2β in neural function.

As an SR protein, Tra2β usually function as splicing factors which regulate alternative splicing by influencing the splice site selection in a concentration-dependent manner [29]. Alternative splicing is a mechanism that controls the protein output of eukaryotic genes, representing a major contributor to proteomic diversity [27]. In the human genome, at least 74% of transcripts are alternatively spliced [43]. As an important process in regulation of gene expression, alternative splicing occurs commonly in the nervous system. In the brain, the regulation of splice variants modulates protein expression levels and functions. As a result, it ultimately affects various physiological and pathological events, including learning and memory, neuronal cell recognition, neurotransmission, ion channel function and receptor specificity [44], [45]. Our results add a new aspect regarding the role of alternative splicing in drug-induced brain responses.

The present study showed that splicing factor Tra2β promoted the inclusion of RGS4 exon 6 and increased the levels of variant RGS4-1 mRNA and RGS4 protein. In addition, RIP assay and co-IP assay demonstrated the presence of RGS4 mRNA and two other SR proteins, ASF/SF2 and SRp30c in the Tra2β IP complex. However, whether ASF/SF2 and SRp30c interact with Tra2β when it is bound to RGS4 mRNAs still need further investigation. As a member of SR proteins, in addition to binding to the target mRNAs, Tra2β is known to bind with other splicing factors, thereby contributing to spliceosome assembly and splicing site recognition [32], [33]. It has been reported that Tra2β and SRp30c promote exon 7 inclusive splicing of the SMN2 gene through an interaction with the AG-rich exonic splice enhancer (ESE) on the exon 7 [33]. In this case, SRp30c does not associate directly with SMN2 exon 7, but through an interaction with Tra2β. In addition, Tra2β and ASF/SF2 are able of binding to the exon 10 of the tau gene and they are required for exon 10 inclusive splicing *in vivo* [32]. It is possible that Tra2β may function on RGS4 mRNAs in a similar way as it does in the regulation of SMN2 exon 7 and tau exon 10 alternative splicing. Whether Tra2β recruits ASF/SF2 and/or SRp30c and contributes to spliceosome assembly around the RGS4 mRNA still need further investigation.

However, in our experiment we observed that Tra2β over-expression leaded to the increase in RGS4-1 mRNA level, but not at the expense of RGS4-4. It is confusing because if Tra2β is stabilizing the inclusion of exon 6, then there should be a decrease in RGS4-4 level. A possible reason is that RGS4-4 mRNAs are unstable and very easy to decay, therefore it is difficult to detect such a low amount of RGS4-4 in RT-PCR, and even much more difficult to detect the decrease of RGS4-4. This possibility is supported by our observation that when the coding sequence of RGS4-4 were inserted in eukaryotic expression plasmids (pAAV and pcDNA3) and transfected into SH-SY5Y cells and HEK293 cells, no translation products were detected by either anti-FLAG antibody or anti-RGS4 antibody, no matter the FLAG tag was located at the N- or C- terminus (data not shown). Besides this, it is also possible that Tra2β increases the level of a RGS4-regulating transcription factor, or simply associates preferentially with RGS4-1 and increases its half-life. This possibility is supported by recent findings that a subset of SR proteins shuttle continuously from the nucleus to cytoplasm, playing important roles in facilitating mRNA transport across the nuclear pore, or having cytoplasmic functions, such as translational regulation, mRNA stability and mRNA localization [46]–[49]. A recently published work of our lab also reported the cytoplasmic localization of Tra2β, suggesting a possible role of Tra2β in cytoplasmic function [50].

The locus coeruleus (LC) is the largest noradrenergic nucleus of the brain. Following chronic morphine treatment, LC neurons show reduced μ-opioid receptor signaling and upregulation of the cAMP pathway, which has been related directly to the dependence and withdrawal syndrome [51]. The reported ability of RGS proteins to negatively modulate Gαi/o function and the upregulation of RGS4 in LC neurons after chronic opiates administration could be a mechanism of μ-opioid receptor desensitization. Therefore, LC represents a useful model system to study chronic actions of opiates in the nervous system. Our dual-labelling experiments showed that Tra2β is highly expressed in TH-positive cells of the LC, adding support to our hypothesis that Tra2β could be involved in morphine-induced functional changes in LC noradrenergic neurons. To assess a potential role for Tra2β in long-term adaptations to morphine administration, we studied the changes in Tra2β protein levels after chronic morphine and naloxone-precipitated withdrawal in the LC. Tra2β immune-reactivity was increased by chronic morphine, and rapidly returned to control levels 2 h after naloxone-precipitated withdrawal. The rapid decreases of Tra2β and RGS4 protein level after acute morphine (3 h) and naloxone-induced withdrawal (1 h) may due to protein degradation processes, which need further investigation. The results show highly dynamic changes in Tra2β expression in the LC, suggesting a potential role of Tra2β in adaptations to chronic morphine exposure.

The molecular mechanisms by which morphine regulates Tra2β expression and function are unknown. *In vivo*, morphine as opiate drug acts primarily on the Gαi/o protein coupled μ-opioid receptor. Activation of Gαi/o protein by μ-opioid receptor leads to several events, including inhibition of cAMP and reduced protein phosphorylation [52]. On the other hand, chronic morphine-induced dependence is a result of adaptation in GPCR signaling in the brain, including super-activation of the cAMP system and changes in PKA and ERK phosphorylation [51], [53]. These events may affect the activity of SR protein phosphatases and/or kinases and provide possible mechanisms that help to explain how Tra2β activity is regulated by morphine. A recent research reported that a component of PKA-dependent signaling pathways, DARPP-32 (dopamine and cAMP regulated phosphoprotein, 32 kDa), competed with protein phosphatase 1 (PP1) to bind to Tra2β and changed the usage of Tra2β dependent alternative exons [54]. In addition, nuclear accumulation of DARPP-32 was promoted by drugs of abuse [55]. Those evidences reveal the connection of cAMP-dependent signaling pathways emanating from the cell membrane with the regulation of nuclear RNA processing.

Our results also showed that besides the LC, Tra2β is co-localized with RGS4 in other brain regions important for opioid actions, such as the PFC, NAc, VTA and PAG. In the central nervous system, the PFC, NAc and VTA belong to mesocorticolimbic dopamine system. It is known that opioids increase dopaminergic signaling, and the increased dopamine signaling underlies the rewarding effects of the opioids and contributes to drug-seeking behavior [56], [57]. The PAG is involved in opioid-induced analgesic responses [58], [59]. Thus, further investigation is needed to fully explore the role of splicing factor Tra2β in opioid actions in these regions.

Moreover, it is worthy to notice that besides RGS4, several other genes show altered RNA processing during opioid action-related events. For example, the μ-opioid receptor gene has 25 splice variants in mice, 8 splice variants in rats and 11 splice variants in humans. The existence of those variants greatly increases the functional diversity and complexity of the μ-opioid receptor gene in agonist-induced G protein activation, adenylyl cyclase activity, receptor internalization and phosphorylation [60], [61]. Many other members of RGS family, including RGS2, RGS3, RGS4, RGS5, RGS6, RGS8, RGS9, RGS12 and RGS19, have been demonstrated to express alternatively spliced mRNA variants. They encode various proteins with different functions in sub-cellular locations and responses to opiate exposure [23], [31], [62]–[70]. Splicing changes in these genes may have a pronounced effect on their roles in the opioid signaling pathway and drug-induced events [60], [71], [72]. However, factors regulating the alternative splicing process of these opioid action-related genes remain unknown. Doubtlessly, exploring the role of SR proteins in regulating the alternative splicing of those genes will provide new insight to understanding the signaling pathway involved in the opioid action-related events.

In conclusion, this study is the first to indicate splicing factor Tra2β as a candidate regulator of brain RGS4 expression in morphine administration. The results provide a novel mechanistic link of alternative splicing to the regulation of opioid-induced signals, adding a new component to the opioid signaling pathway.

## Materials and Methods

### Experimental Animals and Treatments

Male Sprague-Dawley rats (220 to 250 g) from Shanghai Experimental Animal center, Chinese Academy of Sciences, were used in strict accordance with the recommendations in the Guide for the Care and Use of Laboratory Animals of the National Institutes of Health, USA. All rat care and experimental procedures were approved by the Institutional Animal Care and Use Committee of Fudan University Shanghai Medical College (IACUC Animal Project Number: 20070116-xu). Animals were housed three per cage and maintained on a 12 h light-dark cycle. All surgery was performed under chloral hydrate anesthesia and all efforts were made to minimize suffering.

The animals were divided into six groups randomly (6–8 rats for each group) for acute or chronic morphine treatments. Saline (0.9%), morphine (10 mg/kg, Shenyang First Pharmaceutical Factory, China) and naloxone (1 mg/kg, Sigma) were given i.p. This dose of morphine was chosen because it has been found to induce the expression of immediate-early genes by acute administration and elicit the behavioral changes (locomotor sensitization and/or analgesic tolerance) after repeated treatments [20], [73], [74]. In acute morphine and acute saline groups, animals were given a single injection of morphine or equal volume of saline, respectively. In the acute naloxone plus morphine group, animals were given a single injection of naloxone 10 min before the morphine injection. In chronic morphine and chronic saline groups, the chronic drug administration schedule consisted of twice daily injections of morphine (10 mg/kg) or saline in the home cage for 14 days. All animals were sacrificed by rapid anesthesia with 0.36 g/kg chloral hydrate and decapitation at 3 hours after the final injection. Chronic morphine plus naloxone animals were given a single injection of naloxone (1 mg/kg) 2 hours after the final morphine injection on day 14, and were sacrificed 1 hour later.

### Lentiviral Stereotaxic Injection and Cell Infection

Animals were anesthetized with 0.36 g/kg chloral hydrate. Tra2β RNAi lentivirus (1×10^6^ TU/µl, 0.5 µl) was stereotactically delivered into the right LC (coordinates from bregma: AP: −10 mm, ML: +1.4 mm, DV: 6.8 mm from the pial surface). Equal amount of control lentivirus (scrambled siRNA) was stereotactically delivered into the coordinated left side. After recovery from anesthesia, rats were returned to their cages and given *ad libitum* access to food and water for 5 days before sacrificed.

PC12 cells (from ATCC) were cultured in Dulbecco’s modified Eagle’s medium containing 10% fetal bovine serum under 5% CO_2_ at 37°C. Cells plated (at 60% confluence) in 24-well plates were infected with Tra2β RNAi lentivirus or scrambled siRNA lentivirus (1×10^5^ TU/µl, 5 µl) in the medium containing polybrene (5 µg/ml). After 24 hours, the medium containing lentivirus was replaced by fresh medium. Cells were harvested at 3–5 days after infection for further Western blot analysis. Experiments were carried out at least three times from independent cultures.

### Preparation of DNA Constructs

To express the FLAG-tagged Tra2β and FLAG-tagged β-actin, cDNA fragments encoding the full-length human Tra2β protein (NM_004593, 122–988 nt), or the full-length human β-actin protein (NM_001101, 85–1212 nt) were inserted in frame into the pcDNA3-FLAG vector (Invitrogen) at BamHI/XhoI sites. GFP-RGS4 minigene was prepared as follows: RGS4 minigene sequences from exon 4 to exon 7 were amplified by PCR using human genomic DNA as templates. The primers for minigene amplification were E4F (exon 4): 5′- TGC AAA AGA TAT GAA ACA TCG GCT A -3′ and RGS4R1 (exon 7): 5′- CGG GTT GAC CAA ATC AAG ATA GA -3′. This RGS4 minigene was firstly inserted into pMD18-T vectors (Takara), then digested out by *Sac* I and *Sal* I, and cloned in frame with the C-terminal of GFP in pEGFP-C2 vectors (Clontech). For Tra2β RNAi construct, the shRNA sequence targeting 474–494 nucleotides of Tra2β coding sequence were packaged into a pFUGW-RNAi lentiviral vector by Shanghai Genechem Co. Ltd., where the RNAi was driven by the U6 promoter and GFP was driven by human ubiquitin C promoter. All the constructs were confirmed by sequencing.

### Cell Culture and DNA Transfection

Human neuroblastoma SH-SY5Y cells and rat PC12 cells (both from ATCC) were cultured in Dulbecco’s modified Eagle’s medium containing 10% fetal bovine serum under 5% CO_2_ at 37°C. Transfection or co-transfection was performed with FuGENE HD Transfection Reagent (Roche) following the supplier’s protocol. To examine the effects of Tra2β on GFP-RGS4 minigene splicing, SH-SY5Y cells were co-transfected with pcDNA3-FLAG-Tra2β (or empty pcDNA3-FLAG as control) and pEGFP-RGS4 minigene. To examine the effects of Tra2β over-expression on RGS4 expression, SH-SY5Y cells were transfected with pcDNA3-FLAG-Tra2β or empty pcDNA3-FLAG (control). To examine the effect of Tra2β silence on RGS4 expression, SH-SY5Y cells were transfected with double-stranded siRNA oligonucleotides against Tra2β (ON-TARGETplus SMARTpool, Dharmacon) or control non-targeting siRNAs (siGENOME Non-Targeting siRNA#1, Dharmacon). Cells were harvested 48 hours after transfection for RT-PCR or Western blot analysis.

### Reverse Transcription (RT) and Real-time PCR

Total RNA was extracted from the cultured SH-SY5Y cells or RIP samples using TRIzol reagent (Invitrogen) following the supplier’s instructions. Reverse transcription was performed using M-MLV Reverse Transcriptase (Promega) with oligo dT primers. The primers for PCR amplification were as follows: RGS4 forward, 5′- CAA GCC GGA ACA TGC TAG AG -3′; RGS4 reverse, 5′- CGG GTT GAC CAA ATC AAG ATA GA -3′; GAPDH forward, 5′- CAA CAG CCT CAA GAT CAT CAG C -3′; GAPDH reverse, 5′- CAT GAG TCC TTC CAC GAT ACC A -3′; tau forward, 5′- GAG TCC AGT CGA AGA TTG GGT -3′; tau reverse, 5′- GGC GAG TCT ACC ATG TCG ATG -3′.

Real-time PCR was carried out using the SYBR Green Real-time PCR system (TOYOBO) on a LightCycler 480 (Roche Applied Science). The primers were the following: RGS4-1 I2 forward, 5′- ATG CGT CAG TCT TTT CTT CCT CAT CTC TT -3′; RGS4-1 I3 reverse, 5′- AGC CCT TTG CAC ATC TTA TTT -3′, according to the primers reported before [23]; RGS4-4 forward, 5′- GAT ATG AAA CAT CGG CTA GGT -3′; RGS4-4 reverse, 5′- TCC AGG TTC ACA TTC ATG ACT -3′; GAPDH forward, 5′- CAA CAG CCT CAA GAT CAT CAG C -3′; GAPDH reverse, 5′- CAT GAG TCC TTC CAC GAT ACC A -3′. Each sample was tested in triplicates. Absolute quantification of RGS4-1, RGS4-4 and GAPDH were performed with corresponding plasmid as the standards. The relative levels were quantified by the ratio of RGS4-1 or RGS4-4 isoform to GAPDH mRNAs, then the ratio of control was normalized to 1.

### Protein Sample Preparation and Western Blots

Animals were deeply anaesthetized with 10% chloral hydrate, and brain regions were dissected in ice-cold PBS according to standard rat brain stereotaxic coordinates with the help of a mouse brain slicer matrix (Zivi Instruments). The dissected tissue was rapidly frozen in liquid nitrogen.

Brain tissues or cells were homogenized in ice-cold RIPA buffer containing freshly added protease inhibitor cocktail tablets (Roche) and phosphatase inhibitor cocktail tablets (Roche) and centrifuged at 13,000 ×g for 10 min at 4°C. Each LC from one rat was lysed by 100 µl RIPA buffer. Cells harvested from 60 mm dish (about 3×10^6^ cells, 0.3 mg) were lysed by 200 µl RIPA buffer. The total protein concentration of the supernatant was assayed using the BCA assay (Pierce). 10–30 µg of total protein samples were loaded per lane, then separated by 12% SDS-PAGE and transferred to PVDF membranes. The membrane was cut horizontally into several pieces in order to detect proteins of different molecular weights from the same lane. The following antibodies were used: rabbit anti-Tra2β (1∶500, Sigma, S4070), rabbit anti-RGS4 (1∶500, Abcam, ab9964), mouse monoclonal anti-GFP (1∶5000, Millipore, MAB3580), rabbit anti-FLAG (1∶2000, Sigma, F7425), rabbit anti SF2 (1∶500, Abcam, ab38017), rabbit anti-SFRS9 (1∶200, Abcam, ab74782) or mouse monoclonal anti-GAPDH (1∶5000, Kangcheng, Shanghai, KC-5G4). HRP-conjugated secondary antibodies (Kangcheng, Shanghai) were used at 1∶2000. Signals were developed using enhanced chemiluminescence (ECL) substrate (Pierce) onto X-ray films (Kodak). Different exposure times (30 s to 10 min) were used for each membrane to avoid over-exposure of the signals. The immunodensity values of each protein were analyzed by Quantity One 1-D Analysis software (Biorad), and the relative expression levels of each protein were expressed as the ratio of density of each protein to the correspondent GAPDH in the same lane, followed by normalization to the control of each group. Data were obtained from at least three experiments for each condition. The numbers of samples (n) were indicated inside the bar graphs for each figure. The specificity of the anti-RGS4 antibody in immunoblot was confirmed by the experiments shown in Fig. S1.

### RNA-binding Protein Immunoprecipitation (RIP) Assays and Co-immunoprecipitation (co-IP) Assays

For RIP assays, lysates extracted from whole rat brain were immunoprecipitated with rabbit anti-Tra2β antibody (Sigma, S4070) or rabbit anti-GAP43 antibody as negative control (GAP43 protein has the same molecular weight as Tra2β but has no RNA-binding domain) as previously described [75]. Briefly, equal amounts of lysates [about 600 µg/200 µl per RIP reaction, lysed with RIPA buffer with protease inhibitor cocktail (Roche), phosphatase inhibitor cocktail (Roche) and ribonuclease inhibitor (1 U/µl, Takara)] were incubated with antibodies (4 µl antibodies per reaction) and rocking for 2 hours at 4°C and then incubated with Protein A Sepharose beads (20 µl per RIP reaction, Zymed) which were pre-washed in washing buffer (50 mM Tris, pH7.4, 150 mM NaCl, 1 mM EDTA, 10 mM NaF, 1 mM Na_3_VO_4_, 1 mM DTT, 1 U/µl ribonuclease inhibitor and protease inhibitors). After rocking for 1 hour at 4°C, beads were centrifuged at 500 ×g for 5 min at 4°C, then the pellets (beads) were washed five times in washing buffer, with a 5-min rocking interval between each washing. After washing, the RIP beads were then used for RNA and protein extraction (IP). 10% volume of tissue lysate (10% Lysate) and 10% of the supernatants after immunoprecipitation (Sup) were also used for further RT-PCR and Western blot analysis. Experiments were carried out at least three times from independent whole brain lysates.

For RIP with the lysates extracted from SH-SY5Y cells expressing FLAG-Tra2β, FLAG-β-actin (negative control) or FLAG alone (negative control), equal amounts of cell lysates [about 200–400 µg/200 µl per RIP reaction, lysed with RIPA buffer with protease inhibitor cocktail (Roche), phosphatase inhibitor cocktail (Roche) and ribonuclease inhibitor (1 U/µl, Takara)] were incubated with anti-FLAG monoclonal antibody-conjugated agarose beads (5 µl per RIP reaction) and rocking overnight at 4°C. Following the incubation and washing, the cell lysate (10% Lysate) and RIP beads (IP: anti-FLAG) were used for RT-PCR analysis for RGS4 mRNA and tau mRNA (as positive control) and Western blot analysis for FLAG-Tra2β and FLAG-β-actin proteins. Experiments were carried out at least three times from independent cultures.

For co-IP assays, the same cell lysate (10% Lysate) and RIP beads (IP: anti-FLAG) described above were analyzed by SDS-PAGE followed by silver staining or immunoblotting. Peptides sequencing and protein identification were performed by the Mass Spectrometry Core Facility of Institutes of Biomedical Sciences, Fudan University. Experiments were carried out at least three times from independent cultures.

### Immunohistochemical Staining and Fluorescence Immunolabeling

Animals were deeply anaesthetized with 10% chloral hydrate and intracardial perfusion was performed with saline followed by 4% paraformaldehyde. Brains were removed and coronal sections were cut with a freezing microtome (Leica) at a thickness of 30 µm.

For immunohistochemical staining of Tra2β, sections were pre-treated in 0.3% hydrogen peroxide for 20 min to block endogenous peroxidase activity, then were incubated in blocking solution containing 5% BSA and 0.3% Triton X-100 in PBS for 1 hour at 37°C, and subsequently with primary antibodies overnight at 4°C. The following antibodies were used: rabbit anti-Tra2β (1∶500, Sigma, S4070), rabbit anti-RGS4 (1∶500, Abcam, ab9964), mouse monoclonal anti SRp20 (1∶500, Invitrogen, 33–4200). After washes, sections were then incubated with corresponding biotinylated secondary antibodies (1∶200, Vector Laboratories) for 45 min at 37°C, followed by application of avidin-biotin-peroxidase (1∶200, Vectastain Elite ABC kit, Vector Laboratories) for 30 min at 37°C. Immunoreactivity was visualized with 0.05% diaminobenzidine (DAB) (Sigma). Negative controls received the same treatments except that primary antibodies were omitted, and showed no specific staining.

For double fluorescence immunostaining of Tra2β and RGS4, sections were incubated with goat anti-Tra2β antibody (1∶100, Santa Cruz, sc33318) and rabbit anti-RGS4 antibody (1∶500, Abcam, ab9964) at 4°C, overnight. Sections were then incubated with Alexa Fluor (488 or 568 nm)-conjugated secondary antibodies (1∶1000, Invitrogen) at 37°C for 2 hours to reveal the positive signals. The specificity of antibodies in immunofluorescence was confirmed by the blocking experiments shown in Fig. S3.

For triple staining of Tra2β, RGS4 and TH (tyrosin hydroxylase), sections were firstly incubated with goat anti-Tra2β antibody (1∶100, Santa Cruz, sc33318) overnight and then with the corresponding Alexa Fluor (488 nm)-conjugated anti-goat secondary antibody. After rinsing in PBS, the sections were incubated with rabbit anti-RGS4 antibody (1∶500, Abcam, ab9964) and mouse monoclonal anti-TH (1∶500, Sigma, T2928) antibody overnight and then with corresponding Alexa Fluor-conjugated donkey anti-rabbit (568 nm) and donkey anti-mouse (633 nm) secondary antibodies.

After washing, sections with fluorescence immunolabeling were mounted on glass slides and coverslipped using fluoromount medium (Sigma). Fluorescence was detected by confocal laser-scanning microscopy (Leica TCS SP8, Germany).

### Data Quantification and Statistical Analysis

For stereotaxic measurements of the densities of RGS4, Tra2β or SRp20-positive cells in the LC, immunostained sections were observed under a light microscope and immunostained cells were counted in each section using an image-processing and analysis system as previously described [76]. Every 4th of LC-containing coronal section was selected (a total of 5 sections per rat). The densities of immunopositive cells in the LC were presented as the number of the immunopositive cells per cubic millimeter. Slices were coded, and analyzed blindly. Groups of saline, morphine or naloxone treated rat were co-processed for all steps of the protocol.

For all experiments, statistical analyses were performed using two-tailed unpaired Student’s *t* test (for two conditions) or paired *t*-test (for paired samples in immunoblots), or one-way ANOVA followed by Tukey’s multiple-comparison test (for three or more conditions). Data were expressed as means ± SEM from at least three independent experiments. All results statistically different from the control are marked. *p<0.05, **p<0.01, ***p<0.001.

## Supporting Information

Figure S1 **The specificity of anti-RGS4 antibody in immunoblot.** (A) In HEK293 cell which has no endogenous RGS4 expression, both anti-FLAG antibody and anti-RGS4 antibody efficiently recognized the overexpressed RGS4 protein tagged with 3×FLAG repeats at C-terminal (RGS4-3XFLAG, 32 kDa) or one FLAG at N-terminal (RGS4-FLAG, 29 kDa). (B) In SH-SY5Y cell which has endogenous RGS4 expression, anti-RGS4 antibody recognized a strong 28 kDa band and a weak 26 kDa band in mock-treated cells (lane 2),which were indicated by an arrow and an arrowhead, respectively. The molecular weights of these two bands were exactly the same as exogenous overexpressed RGS4 proteins (without any tag, lane 1). In addition, these two bands disappeared when the primary anti-RGS4 antibody was pre-blocked with excess amount of eukaryotic expressed and purified RGS4-3xFLAG protein (10 fold over the antibody, lane 3).
(TIF)

Figure S2 **Effects of Tra2β over-expression and silence on RGS9-1 and RGS9-2 expression in SH-SY5Y cultured cells.** (A) Tra2β over-expression had no obvious effect on relative mRNA level (%GAPDH) of RGS9-1 isoform or RGS9-2 isoform. (B) Tra2β RNAi had no obvious effect on relative mRNA level (%GAPDH) of RGS9-1 isoform or RGS9-2 isoform.
(TIF)

Figure S3 **The specificity of anti-Tra2β antibody and anti-RGS4 antibody in immunofluorescence.** (A, C) Representative immunofluorescence images of goat anti-Tra2β (Santa Cruz, sc33318) and rabbit anti-Tra2β (Sigma, S4070) in rat cortex. (B, D) The immunofluorescence signals of goat anti-Tra2β and rabbit anti-Tra2β antibodies disappeared when the primary antibodies were pre-blocked with excess amount of antigen (prokaryotic expressed and purified GST-Tra2β protein, 50 fold over the antibody). (E) Representative immunofluorescence images of rabbit anti-RGS4 (Abcam, ab9964) in rat cortex. (F) The immunofluorescence signal of rabbit anti-RGS4 (Abcam, ab9964) disappeared when the primary antibodies were pre-blocked with excess amount of antigen (eukaryotic expressed and purified RGS4-3xFLAG protein, 10 fold over the antibody). Scale bars: 80 µm.
(TIF)

Figure S4 **The expression of Tra2β and RGS4 are predominantly in neurons but not in glia.** (A) Representative immunofluorescence images of Tra2β (green), neuronal marker NeuN (red), astrocyte marker GFAP (blue) and their overlay (MERGE) in rat cortex. (B) Representative immunofluorescence images of RGS4 (green), NeuN (red) and their overlay (MERGE) in rat cortex. (C) Representative immunofluorescence images of RGS4 (green), GFAP (red) and their overlay (MERGE) in rat cortex. Scale bars: 80 µm.
(TIF)
