# Supplementary figures and images for: Splicing Factor Transformer-2β (Tra2β) Regulates the Expression of Regulator of G Protein Signaling 4 (RGS4) Gene and Is Induced by Morphine
Source: PLoS One. 2013 Aug 19;8(8):e72220. doi: 10.1371/journal.pone.0072220 (PMC3747076; doi:10.1371/journal.pone.0072220)

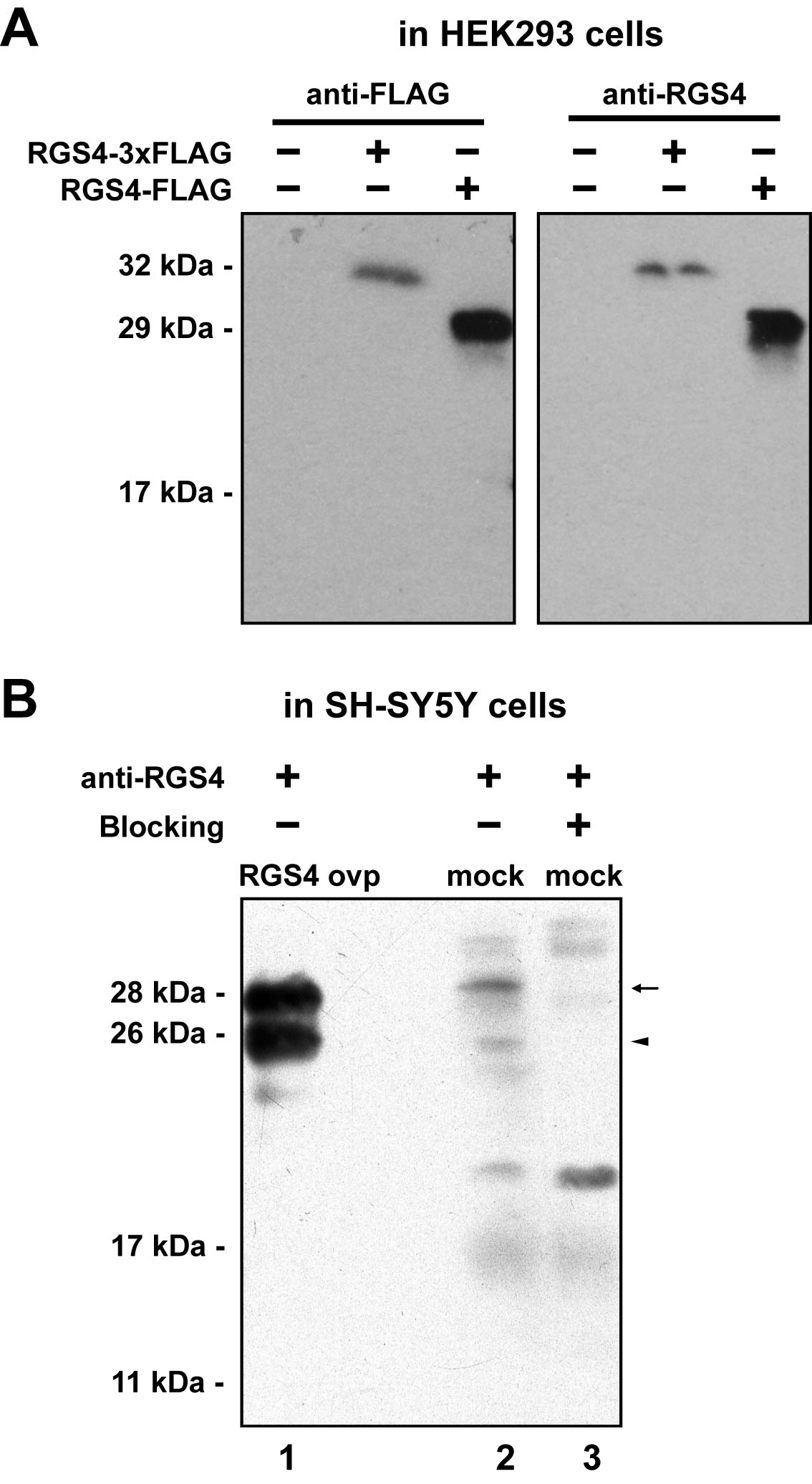

Supplement: Figure S1 — The specificity of anti-RGS4 antibody in immunoblot. (A) In HEK293 cell which has no endogenous RGS4 expression, both anti-FLAG antibody and anti-RGS4 antibody efficiently recognized the overexpressed RGS4 protein tagged with 3×FLAG repeats at C-terminal (RGS4-3XFLAG, 32 kDa) or one FLAG at N-terminal (RGS4-FLAG, 29 kDa). (B) In SH-SY5Y cell which has endogenous RGS4 expression, anti-RGS4 antibody recognized a strong 28 kDa band and a weak 26 kDa band in mock-treated cells (lane 2),which were indicated by an arrow and an arrowhead, respectively. The molecular weights of these two bands were exactly the same as exogenous overexpressed RGS4 proteins (without any tag, lane 1). In addition, these two bands disappeared when the primary anti-RGS4 antibody was pre-blocked with excess amount of eukaryotic expressed and purified RGS4-3xFLAG protein (10 fold over the antibody, lane 3). (TIF) [file pone.0072220.s001.tif]

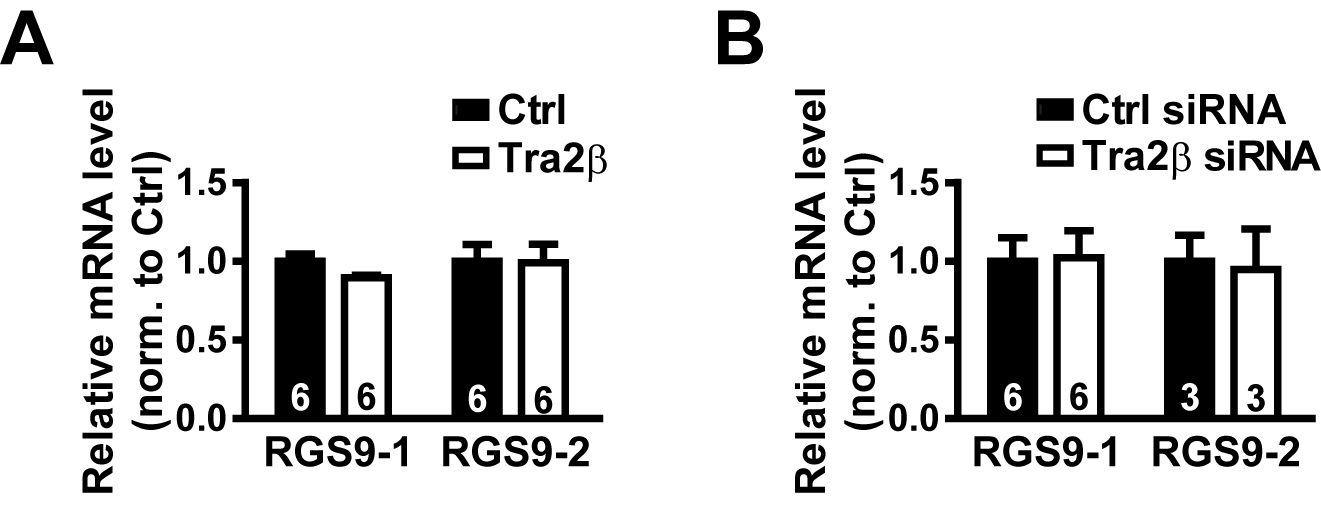

Supplement: Figure S2 — Effects of Tra2β over-expression and silence on RGS9-1 and RGS9-2 expression in SH-SY5Y cultured cells. (A) Tra2β over-expression had no obvious effect on relative mRNA level (%GAPDH) of RGS9-1 isoform or RGS9-2 isoform. (B) Tra2β RNAi had no obvious effect on relative mRNA level (%GAPDH) of RGS9-1 isoform or RGS9-2 isoform. (TIF) [file pone.0072220.s002.tif]

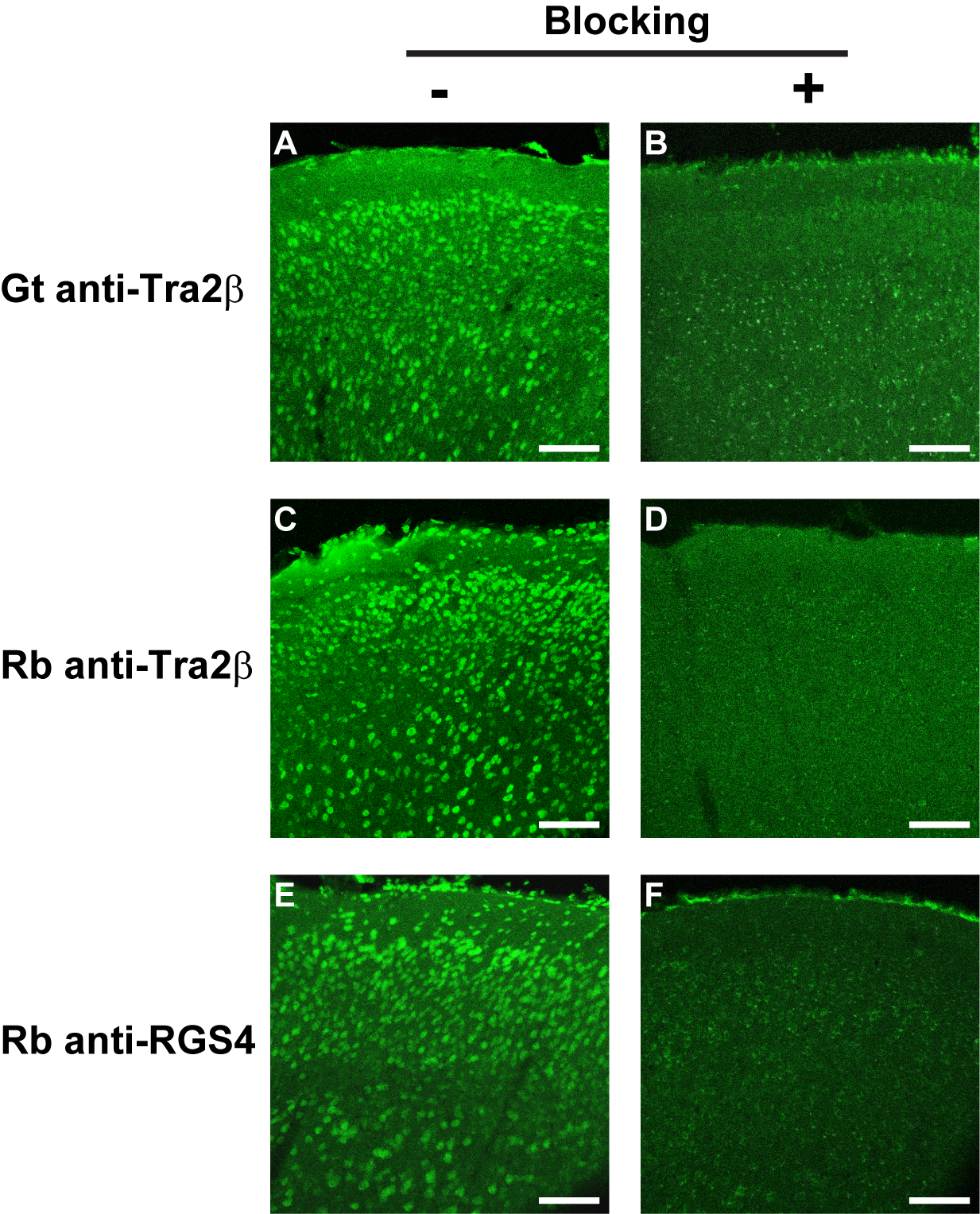

Supplement: Figure S3 — The specificity of anti-Tra2β antibody and anti-RGS4 antibody in immunofluorescence. (A, C) Representative immunofluorescence images of goat anti-Tra2β (Santa Cruz, sc33318) and rabbit anti-Tra2β (Sigma, S4070) in rat cortex. (B, D) The immunofluorescence signals of goat anti-Tra2β and rabbit anti-Tra2β antibodies disappeared when the primary antibodies were pre-blocked with excess amount of antigen (prokaryotic expressed and purified GST-Tra2β protein, 50 fold over the antibody). (E) Representative immunofluorescence images of rabbit anti-RGS4 (Abcam, ab9964) in rat cortex. (F) The immunofluorescence signal of rabbit anti-RGS4 (Abcam, ab9964) disappeared when the primary antibodies were pre-blocked with excess amount of antigen (eukaryotic expressed and purified RGS4-3xFLAG protein, 10 fold over the antibody). Scale bars: 80 µm. (TIF) [file pone.0072220.s003.tif]

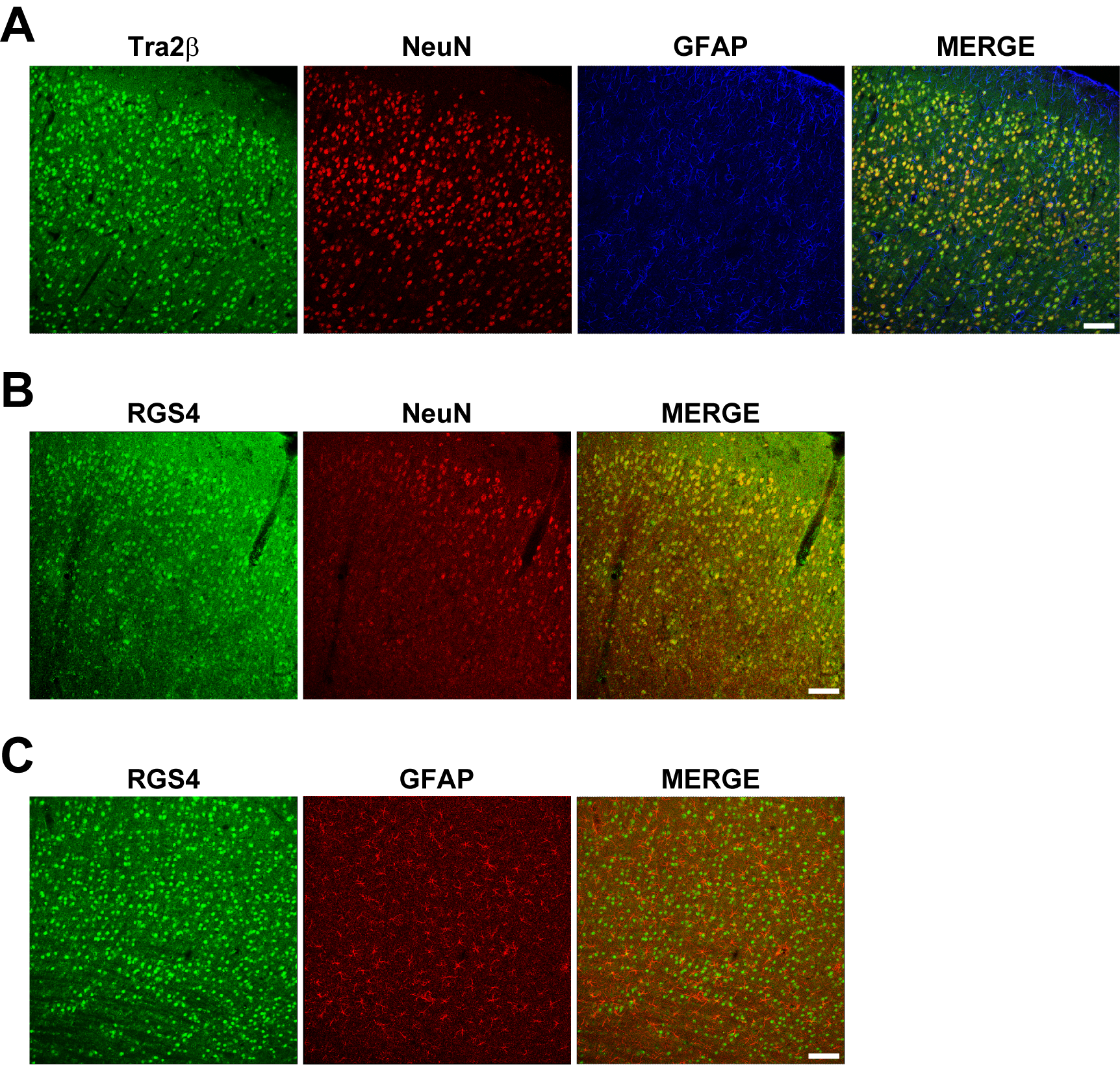

Supplement: Figure S4 — The expression of Tra2β and RGS4 are predominantly in neurons but not in glia. (A) Representative immunofluorescence images of Tra2β (green), neuronal marker NeuN (red), astrocyte marker GFAP (blue) and their overlay (MERGE) in rat cortex. (B) Representative immunofluorescence images of RGS4 (green), NeuN (red) and their overlay (MERGE) in rat cortex. (C) Representative immunofluorescence images of RGS4 (green), GFAP (red) and their overlay (MERGE) in rat cortex. Scale bars: 80 µm. (TIF) [file pone.0072220.s004.tif]
